# Alteration of gut microbiota after heat acclimation may reduce organ damage by regulating immune factors during heat stress

**DOI:** 10.3389/fmicb.2023.1114233

**Published:** 2023-02-23

**Authors:** Shanshou Liu, Dongqing Wen, Chongyang Feng, Chaoping Yu, Zhao Gu, Liping Wang, Zhixiang Zhang, Wenpeng Li, Shuwen Wu, Yitian Liu, Chujun Duan, Ran Zhuang, Lihao Xue

**Affiliations:** ^1^Department of Immunology, Fourth Military Medical University, Xi’an, Shaanxi, China; ^2^Air Force Medical Center, Fourth Military Medical University, Beijing, China

**Keywords:** heat acclimation, heat tolerance test, core temperature, gut microbes, cytokines

## Abstract

**Introduction:**

Heat-related illnesses can lead to morbidity, which are anticipated to increase frequency with predictions of increased global surface temperatures and extreme weather events. Although heat acclimation training (HAT) could prevent heat-related diseases, the mechanisms underlying HAT-promoting beneficial changes in organ function, immunity, and gut microbes remain unclear.

**Methods:**

In the current study, we recruited 32 healthy young soldiers and randomly divided them into 4 teams to conduct HATs for 10 days: the equipment-assisted training team at high temperature (HE); the equipment-assisted training team under normal hot weather (NE); the high-intensity interval training team at high temperature (HIIT), and the control team without training. A standard heat tolerance test (HTT) was conducted before (HTT-1st) and after (HTT-2nd) the training to judge whether the participants met the heat acclimation (HA) criteria.

**Results:**

We found that the participants in both HE and NE teams had significantly higher acclimation rates (HA/total population) than whom in the HIIT team. The effects of HAT on the participants of the HE team outperformed that of the NE team. In the HA group, the differences of physiological indicators and plasma organ damage biomarkers (ALT, ALP, creatinine, LDH, α-HBDH and cholinesterase) before and after HTT-2nd were significantly reduced to those during HTT-1st, but the differences of immune factors (IL-10, IL-6, CXCL2, CCL4, CCL5, and CCL11) elevated. The composition, metabolism, and pathogenicity of gut microbes changed significantly, with a decreased proportion of potentially pathogenic bacteria (Escherichia-Shigella and Lactococcus) and increased probiotics (Dorea, Blautia, and Lactobacillus) in the HA group. Training for a longer time in a high temperature and humidity showed beneficial effects for intestinal probiotics.

**Conclusion:**

These findings revealed that pathogenic gut bacteria decrease while probiotics increase following HA, with elevated immune factors and reduced organ damage during heat stress, thereby improving the body’s heat adaption.

## 1. Introduction

With the global warming trend, extreme high-temperature weather has become more common in recent years. In the latest distressing summer of 2022, extreme and dangerous heat waves spread in India, Pakistan, the United States, Europe, and China, and the highest temperature has repeatedly broken the historical extreme (Morrison, 2022). According to the WHO, approximately 1700 premature and avoidable deaths occurred in Spain and Portugal alone (Lancet, 2022). Notably, greenhouse gas emissions impact various climatic hazards of the Earth’s system (e.g., heatwaves, wildfires, droughts, etc.), which can exacerbate human diseases (Mora et al., 2022). The frequency of heat-related illnesses has increased during abnormal warm weather events. Heatwaves were significantly correlated with a 17% increase in mortality risk due to heat stroke (HS) (Ioannou et al., 2022; Liu et al., 2022; Williams and Oh, 2022). Because of the anticipation of more frequent extreme weather events and associated morbidity/mortality, WHO policy briefs and thermal physiologists have agreed that climate change and heat-related illness are urgent public health concerns (Morrison, 2022).

HS is a severe heat-related illness characterized by the central nervous system (CNS) dysfunction, multiorgan failure, and extreme hyperthermia (usually >40.5°C; Epstein and Yanovich, 2019; Morris and Patel, 2022). Elevated core temperature affects organs such as the brain, gastrointestinal tract, liver, and immune system (Epstein and Yanovich, 2019). Patients in the emergency department with a core body temperature of 41°C or higher have a mortality rate of up to 80% (Gauer and Meyers, 2019). Exertional heat illnesses are the major cause of morbidity in high school athletes and certain occupations (e.g., military, work outdoors in construction or mine shafts; Howe and Boden, 2007; Shimizu et al., 2021; Leiva and Church, 2022; Williams and Oh, 2022). In 2021, active component service members of the United States Armed Forces experienced 488 incidents of HS and 1,864 incidents of heat exhaustion (Williams and Oh, 2022).

Heat acclimation training (HAT) can reduce physiological and perceptual strain in response to both passive and exertional heat stress, potentially reducing HS prevalence (Tyler et al., 2016; Gibson et al., 2020; Westwood et al., 2021). By increasing sweating, expanding plasma volume, and decreasing sodium losses, cardiac output and blood pressure are frequently improved (Heathcote et al., 2018). Lorenzo et al. (2010) demonstrated that in highly trained athletes, HAT could be leveraged to augment aerobic performance. Some factors believed to influence heat acclimation (HA) efficacy include temperature and humidity of the environment, daily training time, and training intensity. Some standard methods for HAT include long-distance running, armed march, hot-water post-exercise exercise, pre-exercise sauna exposure, and so on. Although HAT has been explored by several studies, the effectiveness of different elements remains to be determined owing to large methodological differences (Tyler et al., 2016).

Studies have reported the influence of HAT on organ injury and inflammation response to heat stress (Alves et al., 2022). After HAT, biomarkers of organ injury and inflammatory factors showed significant changes, including alpha-tumor necrosis factor (TNF-α), interleukin-6 (IL-6), IL-10, IL-18, intestinal fatty acid-binding protein (I-FABP), neutrophil gelatinase-associated lipocalin (NGAL), heat shock protein 72 (HSP72), and kidney injury molecule-1 (KIM-1) (Dolci et al., 2015; Lee and Thake, 2017; Chapman et al., 2021; Osborne et al., 2021). The pathophysiology of HS is caused, at least in part, by a systemic inflammatory response induced by gastrointestinal microbe leakage into the bloodstream (Ogden et al., 2021). Cao et al. demonstrated that a 28-day HAT improved the structure of the gut microbiome community and the fecal metabolome in rats, providing novel insights into improving the gut microbiome and its functions as a potential mechanism for HA’s protective properties under heat stress (Cao et al., 2022). However, the alterations in human gut microbe composition and function and their influence on HA remain unclear.

In this study, 32 healthy young soldiers with similar physical conditions, living environments, and dietary structures were recruited. After being randomly divided into 4 teams, they underwent 10-day HATs. We compared the heat acclimation rates of participants in different training teams. We analyzed the changes in physiological indicators, essential organ functions, immunity, and the composition and function of gut microbes after HA. We hypothesized that HA may cause beneficial changes in organ functions, immunity, and gut microbes before and after training, and aimed to explore the significant changes in the human body after exercise-induced HA and key elements affecting intestinal flora after HA.

## 2. Materials and methods

### 2.1. Study population and grouping

The study was approved by the Ethical Committee of the First Affiliated Hospital (Xijing Hospital) of the Fourth Military Medical University, with approved numbers KY20212173 and NCT05155358. All procedures relating to human participants were performed following the Declaration of Helsinki. All participants signed an informed consent form. The inclusion criteria were: male military personnel aged 18–35. The exclusion criteria were: participants who smoked, were injured, or had a history of gastrointestinal, lung, heart, or kidney diseases. Finally, 32 healthy male soldiers with an average age of (24.59 ± 5.36) years took part in this study. The participants received training in the same base for nearly 2 years, and their living environments were similar. All participants did not take any drugs 3 months before and during the experiments. The homogenization process was performed for 7 days with the same daily training and lives. Meals were served in the same cafeteria with a relatively fixed menu and no spicy food. Smoking and drinking were prohibited.

### 2.2. Experimental procedure and physiological data monitoring

First, all the participants underwent a heat tolerance test (HTT)-1st and then were equally divided into 4 teams with random number tables to undergo different heat acclimation trainings (HAT): the equipment-assisted training team at high temperature (HE), the equipment-assisted training team under normal hot weather (NE), the high-intensity interval training team at high temperature (HIIT), and the control team with no training. HAT means a period of exercise training at a high temperature and humidity that may enhance sweat rates and lower exercising core temperatures, resulting in a host of beneficial physiological adaptations. Next, the first three training teams performed HAT: except for the adaptive training on the first day, the participants were asked to complete the planned training within a specified time. After 10 days of training, all participants performed HTT-2nd. Notably, no strenuous exercise, no exposure to hot conditions, and no hot baths were allowed for 2 days before the HTT-1st test. All subjects fasted for 2 h before training and wore sportswear. In addition, the subjects were tested twice in the same period, either in the morning or afternoon. Physiological indicators, including heart rate (HR), rectal temperature (Tcore), and finger oxygen saturation (SaO2), were collected during HATs and HTTs. When HR > 180 beat/min or Tcore >39.5°C, the participants were asked to rest for 3–5 min; if they felt discomfort, dizziness, chest tightness, and palpitation, the training would be terminated, and medical services would be offered immediately. In addition, PSI was calculated based on HTT physiological indicators.


$$\begin{array}{l} PSI\;\left(39.5/180\right)=5\;\left(\text{Tcore}\;\max-\text{Tcore}\;0\right)÷\left(39.5-\text{Tcore}\;0\right)\\ \;+5\left(HR\;\max-HR\;0\right)÷\left(180-HR\;0\right) \end{array}$$

Tcore 0 is the initial value of Tcore before HTT, and Tcore max is the maximum value during HTT.

Besides, HTT was performed at a temperature of (34.5 ~ 35.5) °C and (70 ~ 80) % humidity. The participants were required to complete 4 rounds of pedaling training, each for 30 min (25-min exercise and 5-min rest). The training vehicle (cycle ergometer) was set at 75 W, 60 cycles per minute. After HTT, the Borg Scale, a quantitative scale with a score of 6–20, was used to evaluate the fatigue level (Zamuner et al., 2011; Jo and Bilodeau, 2021). On the scale, 6 is “no exertion,” and 20 is “maximal exertion.” A scale more than 13 means obvious symptoms of breathing and fatigue occurring, and more than 17 indicates the need to stop exercising. Heat intolerance was confirmed with the following occurrences during HTT: HR > 150 beat/min, Tcore >38.5°C, neither tends to plateau (Amit et al., 2013). If the subjects had obvious fatigue or discomfort, such as chest tightness, the training should be stopped immediately, and medical care should be provided. Furthermore, HTT was performed in a high-temperature chamber (DYC-1, Aviation Industry Fenglei Ordnance Co., Ltd. Guizhou, China). The participant’s core temperature was detected with a rectal thermistor (CDR-41, Zeda Technology Co., Ltd. Hangzhou, China) inserted 10 cm beyond the anal sphincter. The temperature was displayed continuously and stored automatically. In addition, HR, SBP, DBP, and SaO2 were constantly monitored and recorded using a comprehensive physiological index monitoring system (TE-4000Y (III) H&L Medical Technology Co. Ltd. Beijing, China). Baseline data of physiological indicators were measured after getting up when the participants were recruited, including body mass index (BMI), body weight, body height, systolic blood pressure (SBP), diastolic blood pressure (DBP), HR, SaO2, and Tcore.

### 2.3. Heat acclimation training program

The HE team received moderate-intensity training (HR130–150 beat/min) for 2 h/day in the high-temperature chamber at a temperature of (34.5 ~ 35.5) °C and humidity of (70 ~ 80) %. To begin, the participants warmed up with gymnastics and then used fitness equipment to assist HAT (including a rowing machine, butterfly machine, dumbbell, and pull-up booster). Each training lasted for 10 min, with a 3-min interval in between. The NE team received moderate-intensity training for 2 h/day in the gymnasium at a temperature of (28 ~ 32) °C and humidity of (45 ~ 55) %. The training program of the NE team was the same as that of the HE team. The HIIT team received high-intensity training (HR150–180 beat/min) for 1 h/day in the high-temperature chamber at a temperature of (34.5 ~ 35.5) °C and humidity of (70 ~ 80) %. After 10 min of warming-up training, 3 rounds of HIIT exercise (including 8 fitting movements: jumping jack, kneeling push-up, self-weight squat, plank hand touch shoulder, alternate lunge squat, mountain step, plank side knee lift, lunge jump) were conducted. Each movement lasted for 45 s, followed by a 15-s rest. After each round of exercise, subjects had 10-min rest and started the next round when HR < 120 beat/min. The control team rested indoors most of the time without training, at a temperature of (23 ~ 27) °C and humidity of (35 ~ 45) %.

### 2.4. Criteria for determining heat acclimation

Indicators such as Tcore, HR, and fatigue score in HTT were used to determine whether the participants met the standards of HA. Criteria for determining HA were followed the “Guidelines for Army Heat Acclimation” by the HS prevention and control expert group in China and as follows: ① to successfully complete HTT-2nd without discomfort (fatigue score ≤ 17) and heat intolerance; ② At the 15th minute after HTT-2nd, HR < 100 beat/min, closing to the initial value; and ③ ∆Tcore (HTT-1st) − ∆T core (HTT-2nd) ≥ 0.2°C, ∆Tcore = Tcore max − Tcore 0.

### 2.5. Sample collection and laboratory testing

Before HTT, an initial blood sample was collected using an EDTA.K2 anticoagulant tube. A total of 8 ml of venous blood was taken from the patients’ cubital vein. The initial physiological indicators (SBP, DBP, HR, SaO2, and Tcore) were also measured. After HTT, post-exercise blood samples were collected immediately, and physiological data were measured after 5-min breaking. Twenty-four hours after HTT, blood samples for the recovery period were gathered. The feces were collected in the morning of two HTTs as pre-training and post-training samples. Clean cotton swabs were used to collect fresh fecal samples (5–10 g, no mix of urine, disinfectant, and sewage) into 15 ml sterile test tubes. Furthermore, the contents of organ injury biomarkers in plasma were detected, including alanine aminotransferase (ALT), alkaline phosphatase (AST), alkaline phosphatase (ALP), bilirubin, lactic dehydrogenase (LDH), alpha-hydroxybutyric dehydrogenase (α-HBDH), creatinine, urea nitrogen, cholinesterase, creatine kinase, prothrombin time (PT), activated partial prothrombin time (APTT), international normalized ratio (INR), Na+, K+, white blood cell (WBC), platelet (PLT), and hemoglobin.

### 2.6. Luminex assay

Luminex detection was performed to assess cytokine concentration in plasma using a Human Premixed Multi Analyte Kit (#FCSTM18-22, R&D Systems, Minneapolis, MN, United States). The factors detected were IL-10, IL-1β, IL-8, IL-6, IL-15, TNF-α, chemokine (C-C-motif) ligand 4 (CCL4), CCL5, CCL11, CCL19, chemokine (C-X-C motif) ligand 2 (CXCL2), CXCL10, vascular endothelial growth factor (VEGF), granulocyte-macrophage colony stimulating factor (GMCSF), and TNF-related apoptosis inducing-ligand (TRAIL). Quality control: the standard curve was fitted with 5-parameter logistic; coefficient of variance ≤20%; and recovery ≥80%.

### 2.7. 16s rDNA sequencing

Gut microbiota 16s rDNA sequencing was conducted on Illumina Novaseq 6000 by Gene Denovo Biotechnology Co., Ltd. (Guangzhou, China). Microbial DNAs from fecal samples of participants in different teams were extracted using HiPure Stool DNA Kits (Magen, Guangzhou, China). Through PCR primers 341F (CCTACGGGNGGCWGCAG) and 806R (GGACTACHVGGGTATCTAAT), the 16S rDNA target region of the ribosomal RNA gene was amplified. Amplicons were then extracted from 2% agarose gels and quantified. According to standard protocols, purified amplicons were pooled in equimolar and paired-end sequenced (PE250) on an Illumina platform.

After the raw reads were acquired by sequencing, the low-quality reads were filtered, and the paired-end reads were spliced into tags. After filtration, the clean tag was clustered, and the chimeric tag was removed to obtain effective tag and OTU abundance. Using an RDP classifier with a confidence threshold value of 0.8, representative OTU sequences were classified into organisms by a Bayesian model. Bioinformatic analysis was performed using online platform Omicsmart.^1^

### 2.8. Statistical analysis

Clinical data of all subjects were collected and recorded. GraphPad Prism 9.0 was adopted for statistical analysis and mapping. The data were evaluated using the Shapiro–Wilk test to determine the normal distribution and were expressed as mean ± standard deviation for normal distribution or median with quartile for abnormal distribution. The baseline data of the 4 teams of volunteers were compared by one-way ANOVA. Student’s *t*-test or Mann–Whitney test was used to compare the mean between the 2 different groups. Repeated measurement data were compared using a paired Student’s *t*-test or paired Mann–Whitney rank sum test. The qualitative data were represented by the constituent ratio. Fisher’s exact probability method was used to compare the rates between different groups. The threshold of statistical significance was set as *p* < 0.05.

## 3. Results

Baseline data, including BMI, body weight, body height, SBP, DBP, HR, SaO2, and Tcore, were obtained at the early morning on the first day of training. Among all 32 subjects, the mean of Tcore-rectal was (37.46 ± 0.26) °C, HR was (74.81 ± 6.71) beat/min, BMI was (23.89 ± 2.54) kg/m^2^, and SaO2-finger was (97.5 ± 0.95) %. There was no significant difference between the baseline of all physiological indicators among the four teams (*p* > 0.05; Table 1).

**Table 1:** Basic characteristics of volunteers.

	Control	HIIT	HE	NE	One-way ANOVA	
	(*n* = 8)	(*n* = 8)	(*n* = 8)	(*n* = 8)	*F*	*p*
BMI (kg/m^2^)	23.76 ± 3.37	22.82 ± 2.78	24.78 ± 1.93	24.20 ± 1.86	0.828	0.490
Body weight (kg)	72.58 ± 11.18	67.35 ± 6.76	77.18 ± 6.24	72.66 ± 5.83	2.123	0.120
Body height (cm)	174.63 ± 3.29	172.00 ± 4.21	176.50 ± 3.66	173.25 ± 1.28	2.731	0.063
SBP (mmHg)	126.0 ± 6.16	123.25 ± 9.66	126.63 ± 5.71	126.00 ± 7.39	0.333	0.801
DBP (mmHg)	83.13 ± 4.55	84.50 ± 6.65	80.50 ± 6.16	80.88 ± 4.22	0.952	0.429
HR (beat/min)	78.50 ± 6.93	76.75 ± 7.07	71.88 ± 6.88	72.13 ± 4.02	2.195	0.111
SaO2 (%)	97.50 ± 0.93	97.63 ± 0.92	97.63 ± 1.06	97.25 ± 1.04	0.257	0.856
Tcore (°C)	37.56 ± 0.22	37.40 ± 0.27	37.45 ± 0.27	37.43 ± 0.29	0.590	0.627

BMI, body mass index; SBP, systolic blood pressure; DBP, diastolic blood pressure; HR, heart rate; SaO2, oxygen saturation (finger); Tcore, core temperature (rectal).

### 3.1. HAT improves the body’s heat adaptability

**Figure 1:**
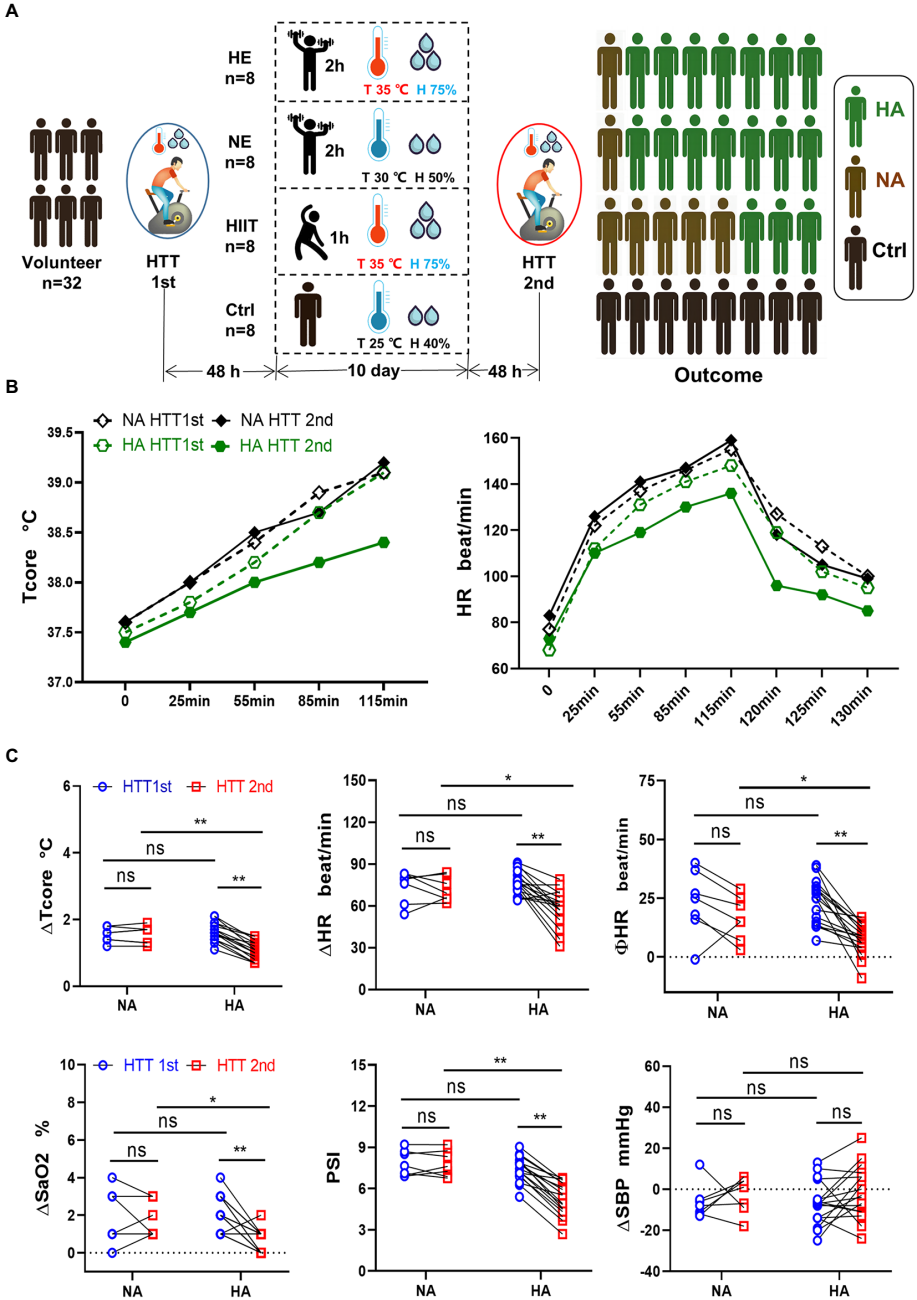
Grouping method, training regimen, HTT testing, and primary outcome. **(A)** Participants were randomly divided into 4 teams and received HTT before and after training. After 10 days of HAT, the acclimation rate of the HE and NE teams was 87.5%, and that of the HIIT team was 37.5%. **(B)** Trend of Tcore and HR of participants in the HA and NA groups during HTT. **(C)** Comparison of physiological indicators between the HA and NA groups during HTT. ΔTcore, ΔHR, ΦHR, ΔSaO2, and PSI of HTT-2nd in the HA group were significantly lower than those of HTT-1st in the HA group and HTT-2nd in the NA group. There was no significant difference in ΔSBP. HA *n* = 17; NA *n* = 7; * *p* < 0.05; ** *p* < 0.01; ns, No significance. HTT, heat tolerance test; HAT, heat acclimation training; Tcore, rectal temperature; H, humidity; HA, heat acclimation; NA, non-heat acclimation; HR, heart rate; SaO2, oxygen saturation (finger); PSI, physiological strain index; SBP, systolic blood pressure; ∆Tcore = Tcore (maximum) − Tcore (initial value); Φ HR = HR (130th min) − HR (initial value).

The data for Tcore and HR during HTT-1st indicated that all 32 participants were heat intolerance and felt tired when pedaling. The data of HTT-2nd revealed that 17 out of the 24 participants in the training teams met the standard of HA (HA group), the other 7 participants were non-HA (NA group). The acclimation rate in the HE and NE teams was 87.5% (7/8), exceeding that of the HIIT team, 37.5% (3/8; Figure 1A). In the control team, significant changes in the physiological data of pre-and post-HTTs were not observed.

We analyzed the changes of the physiological parameters between the HA and NA groups. Tcore and HR values elevated as pedaling time increased, and reached the highest value at 115 min, then gradually decreased after HTT (Figure 1B). In the HA group, ∆Tcore, ∆HR, ∆SaO2, and PSI of HTT-2nd were significantly lower than those of HTT-1st. However, there was no significant change of them in the NA group during 2 times of HTT. No significant difference was found between the HA and NA groups during HTT-1st. In contrast, during HTT-2nd, ∆Tcore, ∆HR, ∆SaO2, and PSI of the HA group were significantly lower than those of the NA group (Figure 1C).

### 3.2. HA is helpful in reducing organ damage and improving the body’s immunity

Immediately after HTT, the plasma content of the organ injury biomarkers increased compared with the initial values and dropped significantly during the recovery period (Supplementary Figures S1A,B). We analyzed the changes in organ damage and immune function caused by heat stress before and after HA by comparing the elevated values of plasma concentrations of the organ injury biomarkers and immune factors.

**Figure 2:**
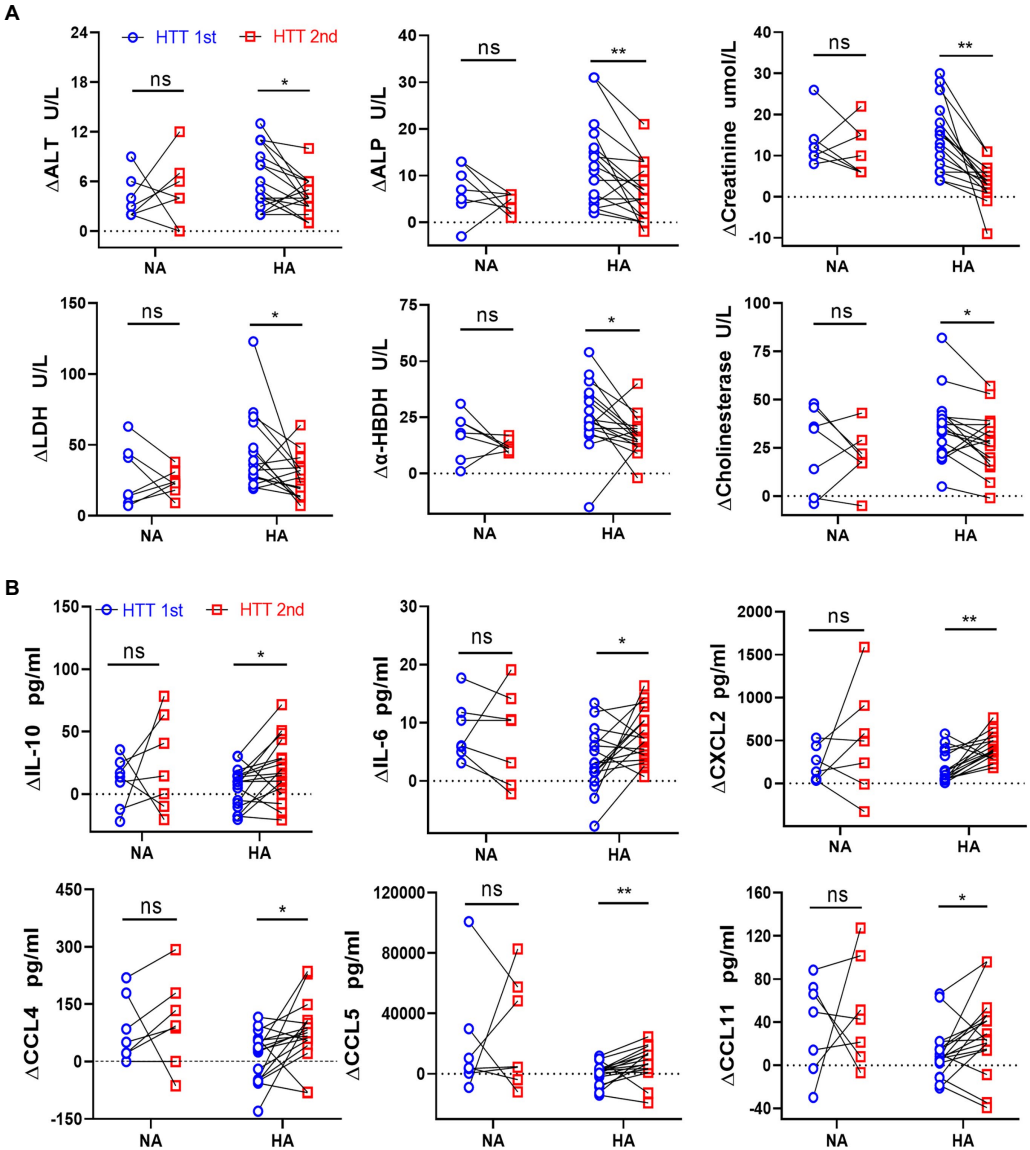
Comparison of the changes in the plasma levels of organ injury biomarkers and cytokines between the HA and NA groups during HTTs. **(A)** Laboratory results: Changes in plasma content of organ injury biomarkers during HTT-2nd were significantly lower than those of HTT-1st after acclimation, including ALT and ALP (liver); creatinine (kidney); LDH and α-HBDH (cardiac and skeletal muscle), and cholinesterase (thermoregulatory center). **(B)** Luminex results: In the HA group, the changes in plasma content of some cytokines of HTT-2nd were significantly bigger than those in HTT-1st, including IL-10 and IL-6 (inflammatory factor) and CXCL2, CCL4, CCL5, and CCL11 (chemokine). In the NA group, there was no significant change in the above molecules during the 2 times of HTT. HA *n* = 17; NA *n* = 7; *p < 0.05; **p < 0.01; ns, No significance. ALT, alanine aminotransferase; ALP, alkaline phosphatase; LDH, lactic dehydrogenase; α-HBDH, alpha-hydroxybutyric dehydrogenase.

In the HA group, the elevated value in the plasma content of some organ injury biomarkers during HTT-2nd was significantly lower than that of HTT-1st, including ALT and ALP (liver); creatinine (kidney); LDH and α-HBDH (heart and skeletal muscle); and cholinesterase (thermoregulatory center; Figure 2A). In contrast, ∆PT, ∆APTT, ∆K^+^, ∆Na^+^, ∆WBC, and ∆PLT did not differ significantly before and after HA (Supplementary Figure S2A).

In the HA group, the elevated values of some cytokine plasma levels during HTT-2nd significantly surpassed those of HTT-1st, including IL-10 and IL-6 (interleukins) and CXCL2, CCL4, CCL5, and CCL11 (chemokines; Figure 2B). Molecules with no difference in plasma content included TNF-α, VEGF, and GM-CSF (cytokine); IL-1β, IL-8, and IL-15 (inflammatory factor); CXCL10 and CCL19 (chemokine); and TRAIL (tumor necrosis factor; Supplementary Figure S2B). However, in the NA group, no significant change was observed in any of the above molecules during 2 times of HTT.

### 3.3. Difference of the gut microbial composition in the HA group before and after training is more remarkable than that in the NA group

**Figure 3:**
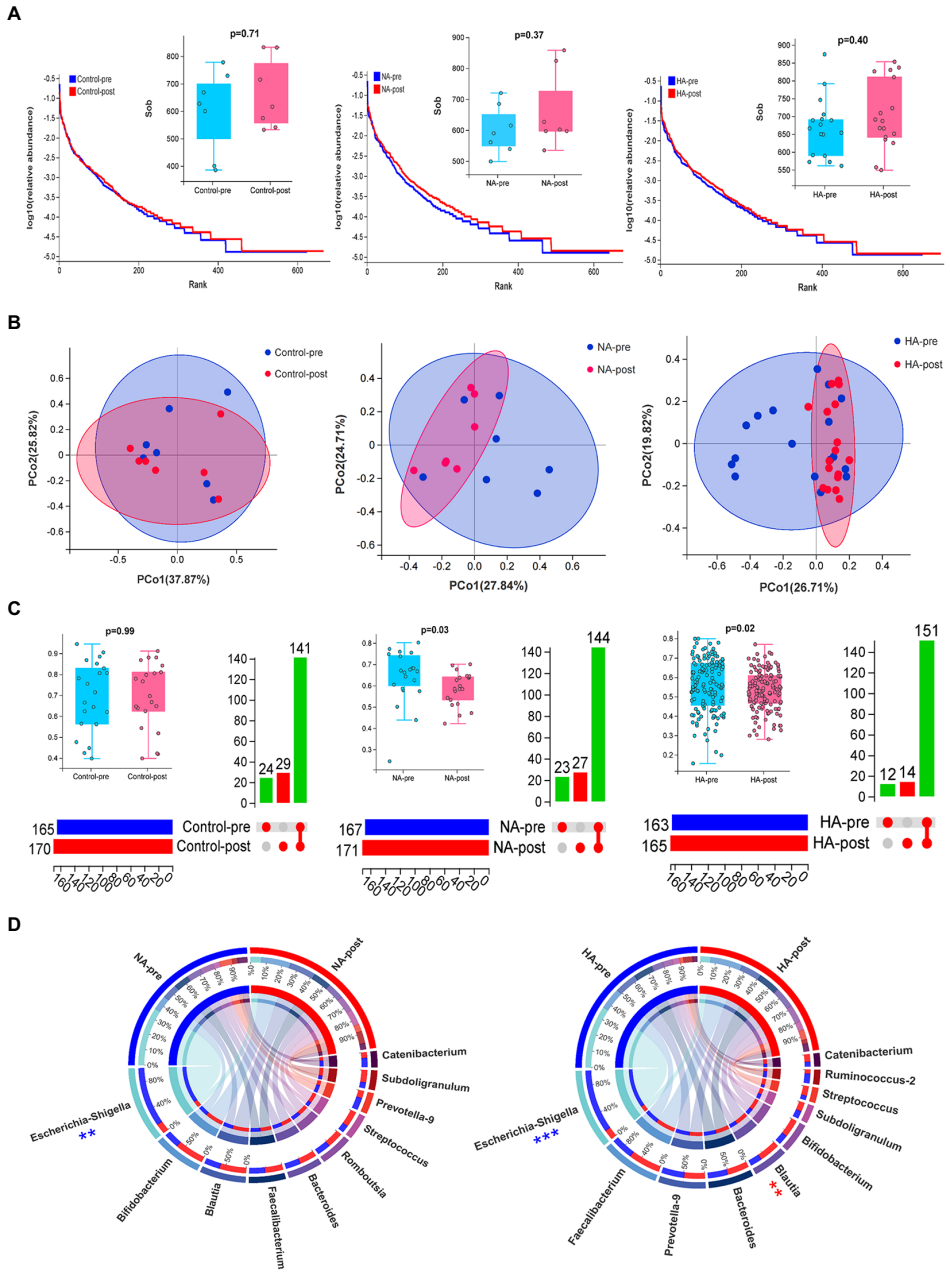
Sequencing results for gut microbiota. **(A)** Alpha diversity analysis: The rank abundance curve of gut microbial abundance, combined with the Wilcoxon test of the SOB index, indicated no significant difference in gut microbial abundance before and after training. **(B,C)** Beta diversity analysis: Differences in the composition of gut microbes were analyzed by PCoA dimension reduction analysis, upset Venn diagram, and Wilcoxon test of bray index. The composition of gut microbes (genus) in the HA and NA groups significantly changed after training. **(D)** Circos diagrams of TOP 10 strains in the HA and NA groups before and after HAT. The proportion of Escherichia-Shigella decreased in both groups, whereas Blautia significantly increased only in the HA group. HA *n* = 17; NA *n* = 7; Control *n* = 7; **p < 0.01; ***p < 0.001.

16s rDNA sequencing analysis of gut microbiota in fecal samples was conducted to analyze how HAT affected the body’s internal environment. The results of the sample quality control indicated that the average effective data ratio exceeded 80%. Most bacterial species in the 2 batch samples were consistent (Supplementary Figures S3A,B). Alpha diversity analysis revealed no significant difference in flora abundance before and after training (Figure 3A).

Beta diversity analysis revealed no difference in flora composition of the 2 batch samples in the control group, but significant differences were observed in the NA and HA groups (Figure 3B,C). Among the top 10 strains with the largest proportion, Escherichia-Shigella significantly decreased after training in the NA and HA groups, while Blautia increased remarkably in the HA group (Figure 3D). The top 10 gut flora before and after training in the control group had no obvious difference (Supplementary Figure S3C).

### 3.4. Indicator species and beneficial changes in bacterial metabolism and pathogenicity after acclimation

**Figure 4:**
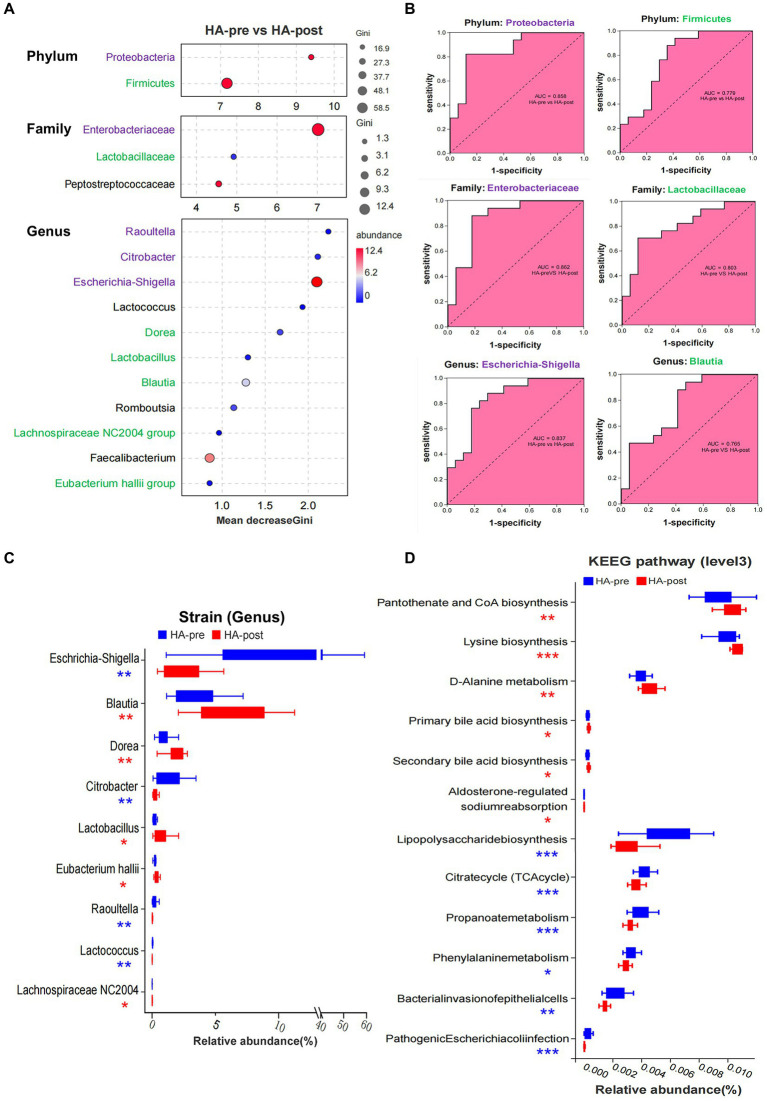
Sequencing results of gut microbiota in the HA group. **(A)** Random forest map of indicator bacteria at phylum, family, and genus levels before and after HAT. Species marked in purple belong to Proteobacteria (phylum), and those marked in green belong to Firmicute. **(B)** ROC of indicator bacteria was used to judge HA. **(C)** Wilcoxon rank sum test was used to analyze the difference in the proportion of bacteria before and after training in the HA groups. **(D)** Wilcoxon rank sum test analysis showed that 12 KEEG pathways were significantly different before and after HAT, mainly involving the synthesis and metabolism of important functional molecules and pathogenicity of bacteria. HA *n* = 17; *p < 0.05; **p < 0.01; ***p < 0.001; ns, No significance. ROC, receiver operating characteristic; HAT, heat acclimation training.

Random Forest was applied to screen potential indicator species. Proteobacteria (phylum) and its branch Enterobacteriaceae (family) had the strongest effects in distinguishing pre-acclimation and post-acclimation, followed by Firmicutes and its branch Lactobacillaceae. At the genus level, strains of Raoultella, Citrobacter, and Escherichia-Shigella, all belonging to Proteobacteria, were top 3 strongest indicators. Blautia, Dorea, Lactobacillus, Lachnospiraceae NC2004 group, and Eubacterium hallii group, as downstream branches of Firmicutes, could also be used as indicators (Figure 4A). We further analyzed the correctness of species as biomarkers using a receiver operating characteristic (ROC) curve. According to the area under the curve of ROC, Escherichia-Shigella and Blautia are the most indicative strains in their respective groups (Figure 4B).

Totally, there were nine strains (genus) that changed significantly after HAT. Four pathogenic strains, such as Escherichia-Shigella, Citrobacter, Raoultella, and Lactococcus, significantly decreased after acclimation. In contrast, Blautia, Dorea, Lactobacillus, Lachnospiraceae NC2004 group, and Eubacterium hallii group significantly increased, all of which were beneficial strains (Figure 4C). However, the significantly changed gut microbiota in the NA group were reduced Escherichia-Shigella and increased Collinsella after training (Supplementary Figure S3D). After acclimation, the proportions of the 7 functional phenotypes changed significantly. Among them, potentially pathogenic bacteria, gram-negative, facultatively anaerobic, oxidative stress tolerant, and biofilm-forming bacteria significantly decreased, whereas gram-positive and anaerobic bacteria increased (Supplementary Figure S3E).

Wilcoxon rank sum test analysis showed that 12 KEEG pathways were significantly different after HA, mainly involving the synthesis and metabolism of critical functional molecules as well as the pathogenicity of bacteria. Signal-enhancing pathways included pantothenate and CoA biosynthesis, lysine biosynthesis, D-Alanine metabolism, primary bile acid biosynthesis, secondary bile acid biosynthesis, and aldosterone-regulated sodium reabsorption. However, signal-reducing pathways included lipopolysaccharide biosynthesis, citrate cycle (TCA cycle), propanoate phenylalanine metabolism, bacterial invasion of epithelial cells, and pathogenic Escherichia coli infection (Figure 4D).

### 3.5. Training for a longer time and higher intensity in a higher temperature and humidity is beneficial for probiotics

**Figure 5:**
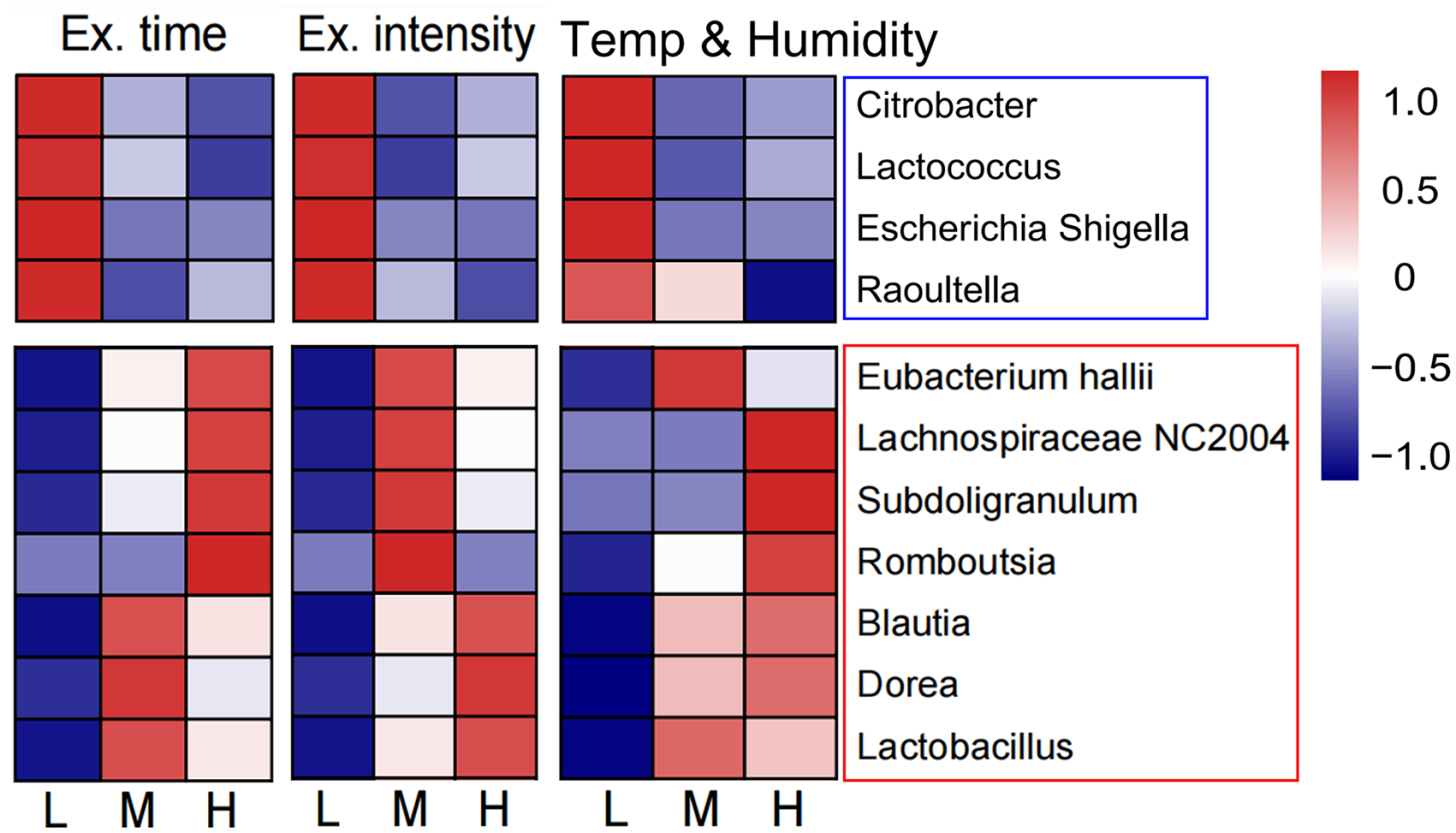
Changes of intestinal flora composition under different training elements. The heat map showed the relationship between three training elements (exercise time per day, exercise intensity and the environmental temperature and humidity) and the abundance of intestinal probiotics and pathogenic bacteria. L, low; M, moderate; H, high.

We also preliminary analyzed the changes in intestinal flora composition under different training factors, including exercise time per day, exercise intensity, and environmental temperature and humidity (Supplementary Table S1). It was found that training for a longer time and higher intensity in a higher temperature and humidity decreased the relative abundance of pathogenic bacteria, such as Escherichia-Shigella, Citrobacter, Raoultella, and Lactococcus. However, under the same training conditions, the proportion of Dorea, Blautia, Lactobacillus, Lachnospiraceae NC2004 group, and Subdoligranulum increased, all of which were probiotics (Figure 5). Therefore, the structure of intestinal flora may present a beneficial change after a longer training and higher intensity in a higher temperature and humidity.

### 3.6. Longer daily training time and higher heat exposure facilitate HA

The acclimation rate in the training teams was 70.83% (17/24). Seven people did not meet the criteria for HA, 5 of whom were in the HIIT team (high humidity, high-intensity but short daily training time). Grouped by daily training time, the participants in both HE and NE teams trained for 2 h/day (although moderate-intensity training under normal heat exposure) had a significantly higher acclimation rate than the participants in the HIIT team trained for 1 h/day [87.5% (14/16) vs. 37.5% (3/8), *p* = 0.021].

**Figure 6:**
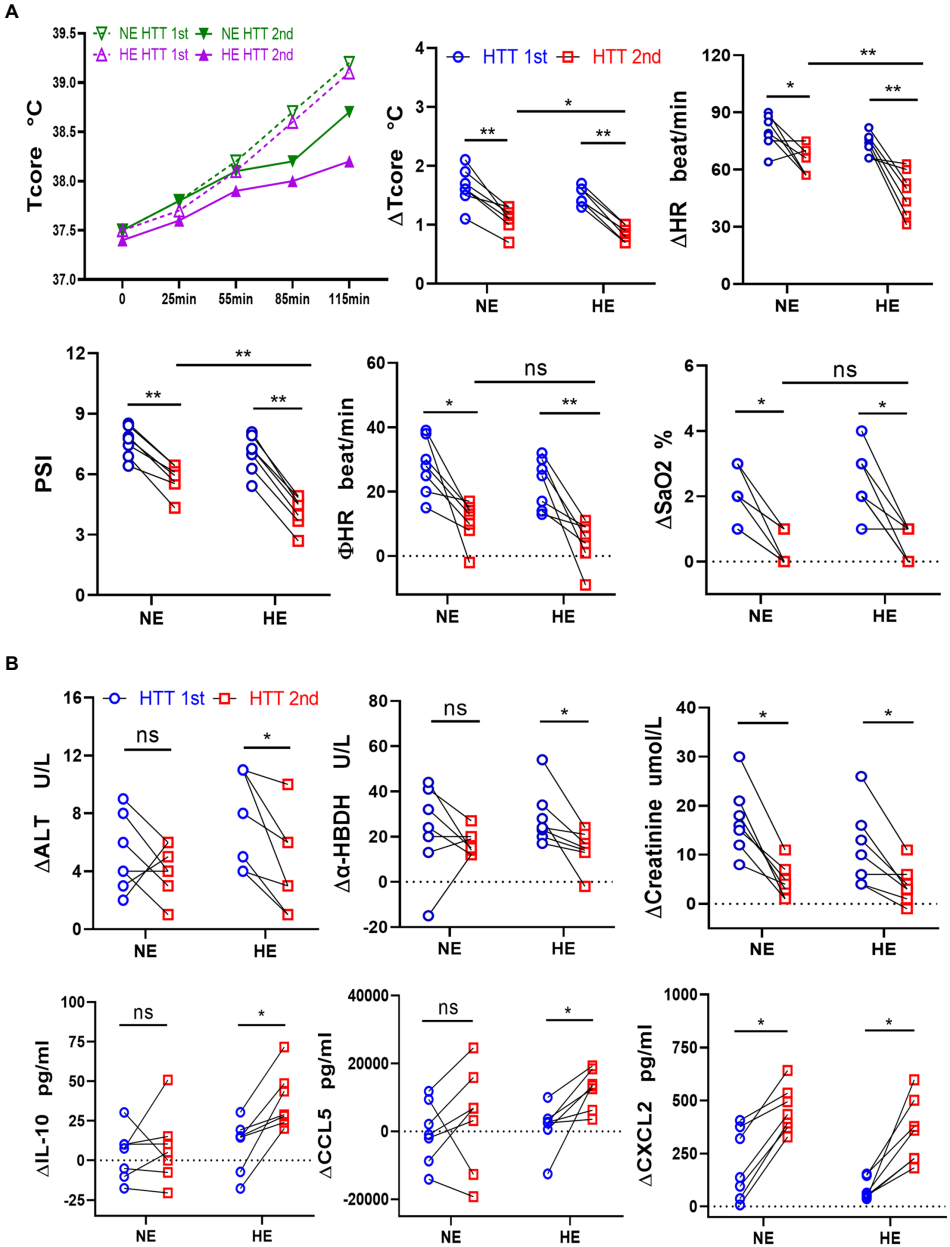
Changes in physiological indicators, plasma levels of organ injury biomarkers, and cytokines during HTT in the HE and NE teams. **(A)** In HTT-2nd, ΔTcore, ΔHR, and PSI of the HE team were significantly lower than those of the NE team, but there was no significant difference in ΦHR and ΔSaO2. **(B)** In the HE team, ΔALT and Δα-HBDH in HTT-2nd were significantly lower than those of HTT-1st, but ΔIL-10 and ΔCCL5 were significantly higher. In the NE team, the indicators also showed the same trend, but the difference was insignificant. HE *n* = 7; NE *n* = 7; *p < 0.05; **p < 0.01; ns, No significance. HTT, heat tolerance test; Tcore, rectal temperature; HR, heart rate; PSI, physiological strain index; SaO2, oxygen saturation (finger); ALT, alanine aminotransferase; α-HBDH, alpha-hydroxybutyric dehydrogenase; ∆Tcore = Tcore (Maximum) − Tcore (initial value); Φ HR = HR (130th min) − HR (initial value).

The acclimation rate was 87.5% in both HE and NE teams, with the same daily training time and training intensity. We further compared the changes in the physiological function, immunity, and gut microbes between HE and NE teams. We found that the HAT effects of the HE team with higher heat exposure outperformed that of the NE team. In HTT-2nd, ΔTcore, ΔHR, and PSI of the HE team were significantly lower than those of the NE team. In HTT-1st, the above indicators did not significantly differ between the 2 teams. All physiological indicators of HTT-2nd after training were significantly lower than those of HTT-1st in the HE and NE teams (Figure 6A). In the HE team, ΔALT and Δα-HBDH in HTT-2nd were significantly lower than those of HTT-1st, and ΔIL-10 and ΔCCL5 were significantly higher. The biomarkers of the participants in the NE team also showed the same trend, but the difference was not significant (Figure 6B).

**Figure 7:**
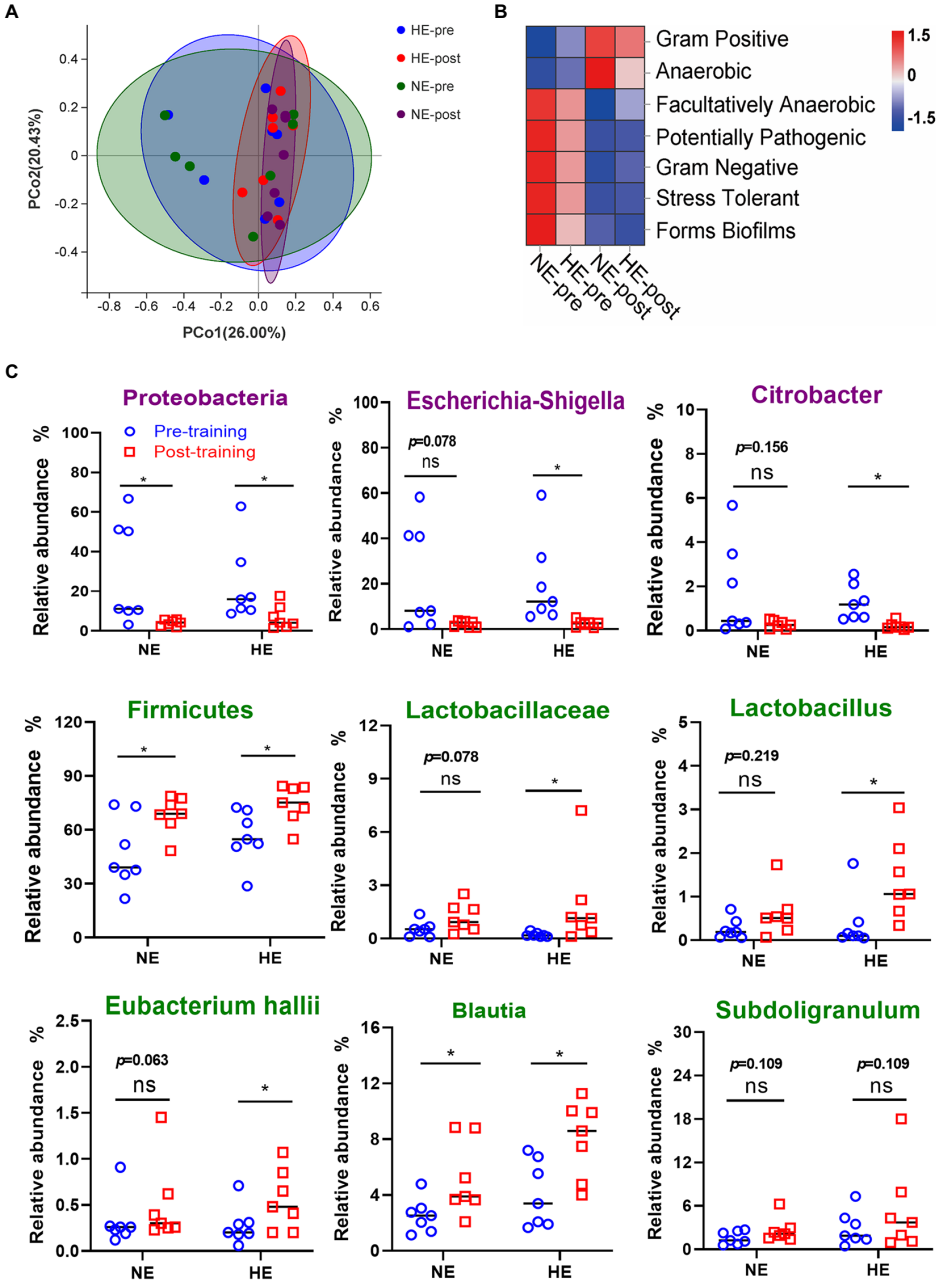
Sequencing results of gut microbiota in the HE and NE teams. **(A)** PCoA dimensionality reduction analysis was used to analyze the changes in gut microbiota composition before and after HAT. **(B)** Changes in the proportion of functional phenotypes in the HE and NE teams before and after HAT. **(C)** Difference in the changes in the proportion of indicated bacterial species. In the HE team, the proportion of pathogenic bacteria (Escherichia-Shigella and Citrobacter) significantly decreased after training, but probiotics (Lactobacillus and Eubacterium hallii groups) increased. In the NE team, the changes in the above strains also showed the same trend, but the difference was not significant. NE *n* = 7; HE *n* = 7; *p < 0.05; ns, No significance.

PCoA dimensionality reduction analysis revealed that the composition of gut microbes in the HE and NE teams changed significantly after HAT (Figure 7A). Seven functional phenotypes showed the same trend as the HA group, among which gram-positive and anaerobic bacteria increased, and the other 5 phenotypes decreased (Figure 7B). In the HE team, after training, the proportion of pathogenic bacteria (Escherichia-Shigella and Citrobacter) was significantly reduced, whereas the ratio of probiotics (Lactobacillus and Eubacterium hallii groups) was significantly increased. The change in gut microbes in the NE team was not significant (Figure 7C).

## 4. Discussion

Global warming projections forecast a 2°C increase in global average temperatures by 2,100, including extreme weather events. Future heat exposures for humans will exceed critical levels more frequently in tropical countries like India, where heat waves have increased in frequency and severity (Morrison, 2022; Weitz et al., 2022). Heat-related illnesses may be mitigated by improved surveillance systems, effective interventions, and prevention strategies (Abasilim and Friedman, 2022). HA had a moderate to large beneficial effect on lowering core body temperature during exercise and maintaining cardiovascular health, which has been reported in gold miners to reduce their mortality in South Africa (Tyler et al., 2016; Schermann et al., 2018). The current project aims to quantify the changes in physiological indexes, organ damage indexes, inflammatory cytokines, and intestinal flora composition before and after the training of HA and NA groups.

We recruited 32 healthy young soldiers who had received the same training subject in the past 2 years. The military training environment was similar, but physical characteristics were not measured. No significant difference was observed in the baseline data of physiological indicators. All participants were homogenized for 7 days and randomly divided into 4 teams. During HAT, their work and rest rhythm, eating, training, sample collection, and indicator measurement were strictly controlled. Data analysis also considered the self-comparison of repeated measurement data within groups to minimize the influence of confounding factors and improve the conclusion’s reliability.

The individualized HTT scheme we adopted is a beneficial attempt to extend the application of HTT. In addition, the comprehensive judging criteria we adopted for HA supplemented the quantitative criteria of key indicators in the “Guidelines.” The current standards for HA are as follows: ① No symptoms of discomfort in a high temperature and high humidity environment, and feeling comfortable; ② HR is close to the initial value within 10–15 min after training; ③ Increased body core temperature decreases; and ④ increase in sweating.

After acclimation, the degree of change in physiological indicators has become relatively mild, which is, of course, logical and realistic. Changes in Tcore and HR during heat exposure are often used to determine HA. Considering the significance of combining thermal and cardiovascular responses to HS, the PSI and the thermal-circulatory ratio (TCR) were also developed as physiological criteria for evaluating individuals who underwent HTT (Moran et al., 2007; Ketko et al., 2014). Physiological strain is rated on a scale of 0–10 using PSI, a valid and simple physiological index under different protocols (Moran et al., 1998; Byrne and Lee, 2019). In the current experiment, we also initially tried to use SaO2. This index comprehensively reflected the function of the respiratory and circulatory systems, to distinguish whether the participants were acclimatized or not. The results showed that the working efficiency of SaO2 was consistent with the other 3 classical indexes. Interestingly, SaO2 monitoring is more advantageous in terms of stability and convenience. This study provided a new indicator for evaluating the HA effects.

After HA, the increased plasma biomarkers (ALT, ALP, creatinine, LDH, α-HBDH, and cholinesterase) was significantly lower than before, suggesting that the organs adapted to heat strain during HAT. Notably, the elevation of LDH was significantly reduced during HTT-2nd, suggesting that the homeostasis of the microcirculation function was enhanced after acclimation. A meta-analysis focusing on the physiological effects of HAT revealed that HA might reduce oxygen consumption and lactate concentrations (Tyler et al., 2016), which corroborates the current study’s findings. In addition, the significantly changed cholinesterase could not only reflect liver injury but also participate in the physiological function of the thermoregulatory center (Wang and Weinstock, 2001; Sun et al., 2007). Donepezil, a centrally acting acetylcholinesterase inhibitor, caused marked bradycardia and hypothermia in conscious mice through muscarinic receptor activation (Polichnowski et al., 2021). This new finding reveals that cholinesterase may be a candidate biomarker for HA and a potential therapeutic target for HS. What’s more, biomarkers of organ injury, such as liver-type fatty acid-binding protein (L-FABP), intestinal fatty acid-binding protein (I-FABP), total protein, albumin, and kidney injury molecule-1 (KIM-1), showed significant changes after HAT (Chapman et al., 2021; Ravanelli et al., 2021).

Inflammatory cytokines, such as CCL5, IL-6, IL-1β, IL-8, TNF-α, INF-γ, and MCP-1, are highly increased in HS patients and animal models, the levels of which correlate with organ failure and a fatal outcome (Lu et al., 2004; Chen et al., 2017). Our data showed that after acclimation, the plasma levels of immune factors (CCL5, IL6, IL-10, CXCL2, CCL4, and CCL11) were significantly higher than before but did not exceed the normal physiological range, which differed from the trend of explosive increase in inflammatory factors in HS. The results confirmed that HTT and HAT in this subject did not cause immune dysfunction and led to the inflammatory injury of organs. Meanwhile, a slight increase in the physiological level of inflammatory factors may help reduce organ damage. IL-6 benefits physiological responses to severe hyperthermia, protecting against organ damage and inflammation (Phillips et al., 2015). In addition, most of the increased interleukin and chemokines were related to macrophages, suggesting that HA may maintain the body’s homeostasis during heat exposure by improving the sensitivity of the innate immune cells, which could kill the invaded pathogens due to physiological barrier destruction. Besides, inflammatory factors concentrations in serum and urine, including alpha-tumor necrosis factor IL-18, neutrophil gelatinase-associated lipocalin (NGAL), and heat shock protein72 (HSP 72) were also reported to be affected by heat acclimatization training (Dolci et al., 2015; Chapman et al., 2021).

The gut is one of the major target organs affected by heat stress, which induces damage to the microstructures of the mucosal epithelia, reduces immunity, and increases gut permeability to toxins and pathogens (He et al., 2019; Patra and Kar, 2021). Our intestinal tract is a nutrient-rich environment with up to 100 trillion microbes (Ali et al., 2020). Changes in body temperature, a core factor that controls microbial growth *via* hypo-and hyperthermia, influence the gut microbiota in various animals, with consistent effects on community diversity and stability (Huus and Ley, 2021). Warming caused changes in the Lizard gut microbiome, including a significant decrease in the relative abundance of Firmicutes (Moeller et al., 2020). While stress reduces the number of Lactobacilli, it increases those of *E. coli* and Pseudomonas (Lutgendorff et al., 2008). Our findings suggest that the changing trend in gut microbiota structure and function after HA is the inverse of that observed after heat stress. After acclimation, the proportion of potentially pathogenic bacteria, such as Escherichia-Shigella, decreased significantly, whereas probiotics, such as Lactobacillus and Blautia, increased significantly. KEGG functional enrichment analysis revealed that bacterial invasion of epithelial cells, lipopolysaccharide biosynthesis, and pathogenic *E. coli* infection were attenuated considerably. In an experimental rat model, gut microbiota composition was altered by HA with significant increases in the genera Lactobacillus (Cao et al., 2022), which was consistent with the current study. The above results suggest that HA training promotes the optimization of intestinal flora and reduces the inflammatory response caused by intestinal infections.

Accumulating studies have shown that the altered microbiome likely orchestrates the body’s various relevant biological phenomena (Patra and Kar, 2021). Evidence has demonstrated that flora exerts fundamental effects on the development of HA, and modulation of gut microbiota improves tolerance to heat exposure and reduces the risks of heat-related illnesses (Cao et al., 2022; Houser et al., 2022). However, the exact mechanisms underlying how functional communications occur between the microbiota and HA responses remain unexplored. The microbiota-gut-brain axis may be altered by heat stress because it adversely affects intestinal immunity, barrier function, and microbiota metabolites (Wen et al., 2021). Probiotics counteract stress-induced changes in intestinal barrier function and mucosal inflammation by regulating macrophage cytokines and NF-κB activation (Lutgendorff et al., 2008; Habil et al., 2011). Our data showed that the proportion of intestinal probiotics significantly increased after HA, and anti-inflammatory factor ∆IL10 in the second time of HTT was obviously higher than the previous one. At the same time, organ damage biomarkers (∆ALT, ∆ALP, ∆LDH, and ∆α-HBDH) decreased, which indicated that probiotics might reduce organ damage during heat exposure by inhibiting inflammation. These findings provide novel insights into the optimization of gut microbiota in composition and function as a potential mechanism by which HA confers heat stress protection.

KEGG analysis of the metabolic function of gut microbiota showed that pathways of aldosterone-regulated sodium reabsorption, primary bile acid biosynthesis, and secondary bile acid biosynthesis were markedly enriched in the HA group after training. Changes in flora structure enhance aldosterone-regulated sodium reabsorption, apparently contributing to maintaining water balance and electrolyte stability during heat exposure. The enhanced synthesis of bile acids may be related to increased probiotics after acclimation. Proteomics has fingerprinted a close relationship between bile acid and Lactobacillus (Ali et al., 2020). Bile acids play a crucial role in regulating intestinal immunity and inflammation, helping maintain intestinal homeostasis. Oral administration of microbiota-derived secondary bile acid sodium deoxycholate to mice reduced C. jejuni-induced colitis (Sun et al., 2018). In addition, bile acid metabolites control host immune responses by promoting the generation of peripheral regulatory T cells, directly modulating the balance of Th17 and Treg cells and gut RORγ^+^ regulatory T cell homeostasis (Hang et al., 2019; Campbell et al., 2020; Song et al., 2020).

The relationship between gut microbiota and HA is complex, and further research is needed to discuss the molecular mechanism underlying how HA affects microbiota composition, mucosal structure, immunity, and barrier integrity in the gut. Here, we preliminary discovered that a longer training time and higher environmental temperature and humidity may contribute to beneficial bacteria changes in gut microbiota. Correspondingly, the acclimation rate of participants in the HE and NE teams trained for 2 h/day significantly exceeded that of the HIIT team trained for 1 h/day. HIIT may improve maximum oxygen consumption, which can burn slightly more body fat than moderate-intensity continuous training (Nayebifar et al., 2016; Abbasi, 2019). When the training time and intensity were consistent, the acclimation effects of the HE participants training under higher heat exposure outperformed that of the NE team, although the acclimation rate did not show an advantage. Therefore, most of the common methods of HAT, such as sauna or heat chamber and hot baths or hot-water immersion post exercise, were used to provide moderate heat stress for the purpose of HA (Heathcote et al., 2018). Accordingly, organizers should consider heat exposure, training time, and intensity when developing the HAT program.

Some limitations exited in this study. For example, the participant size was relatively small, and heterogeneity may still exist though physical condition and diet structure of the participant were similar. Besides, some organ-specific biomarkers had not been measured, e.g., intestinal bile acid binding protein (I-ABP), lipopolysaccharide binding protein (LBP), lactulose, and endotoxin for gut and L-FABP and NGAL for kidney. Moreover, there was no correction of plasma biomarker concentration for changes in plasma volume, and no HS/heat illness cases in the subclinical exertional heat stress test. As extreme high-temperature weather has become more common in recent years, more clinical specimens and experimental data would be considered for further exploration and verification.

## 5. Conclusion

In conclusion, as summer heatwaves become increasingly common owing to increased global warming, the morbidity and mortality from heat-related illnesses will continue to rise. The current study verified that a better exercise performance after HA showed enhanced cardiopulmonary function and decreased indexes of organ damage with altered immune indicators, suggesting an improved body thermal adaptability. In addition, the proportion of probiotics significantly increased, which may reduce organ damage and the expression of inflammatory factors, informing that intestinal flora plays an essential physiological role in HA. This study reveals a close relationship between gut microbiota and HA, which provides novel insights for developing a more scientific and effective HAT scheme.

## Data availability statement

The data presented in the study are deposited in the NCBI Sequence Read Archive (SRA) database, accession number PRJNA909423.

## Ethics statement

The studies involving human participants were reviewed and approved by the Ethical Committee of the First Affiliated Hospital (Xijing Hospital) of the Fourth Military Medical University. The patients/participants provided their written informed consent to participate in this study.

## Author contributions

RZ and LX conceived of the study and participated in its design and coordination. DW, CY, ZG, LW, and ZZ designed and conducted the experiments. SL, CF, WL, SW, and YL acquired the data and performed the statistical analysis. SL and CD drafted the manuscript. LX and RZ helped to revise the manuscript. All authors have read and approved the final version of the manuscript, and agree with the order of presentation of the authors.

## Funding

This work was supported by the Logistic Support Department of PLA [grant number 18QNP026] and the Fourth Military Medical University [grant number 2020rcfczr].

## Conflict of interest

The authors declare that the research was conducted in the absence of any commercial or financial relationships that could be construed as a potential conflict of interest.

## Publisher’s note

All claims expressed in this article are solely those of the authors and do not necessarily represent those of their affiliated organizations, or those of the publisher, the editors and the reviewers. Any product that may be evaluated in this article, or claim that may be made by its manufacturer, is not guaranteed or endorsed by the publisher.

## Footnotes

^1^ http://www.omicsmart.com
