# Supplementary figures and images for: Alteration of gut microbiota after heat acclimation may reduce organ damage by regulating immune factors during heat stress
Source: Front Microbiol. 2023 Feb 23;14:1114233. doi: 10.3389/fmicb.2023.1114233 (PMC9995595; doi:10.3389/fmicb.2023.1114233)

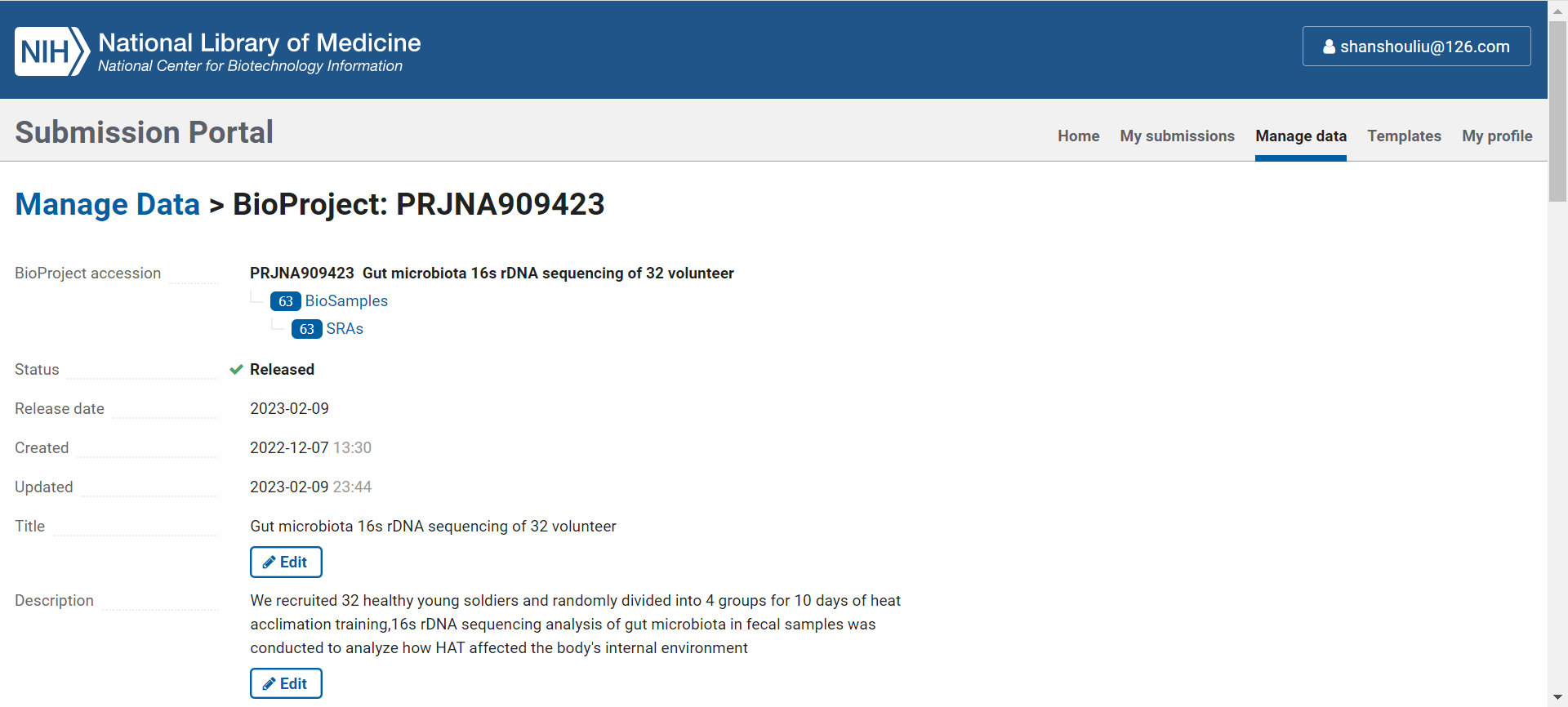

Supplement: Supplementary file 1 [file Image_1.tif]

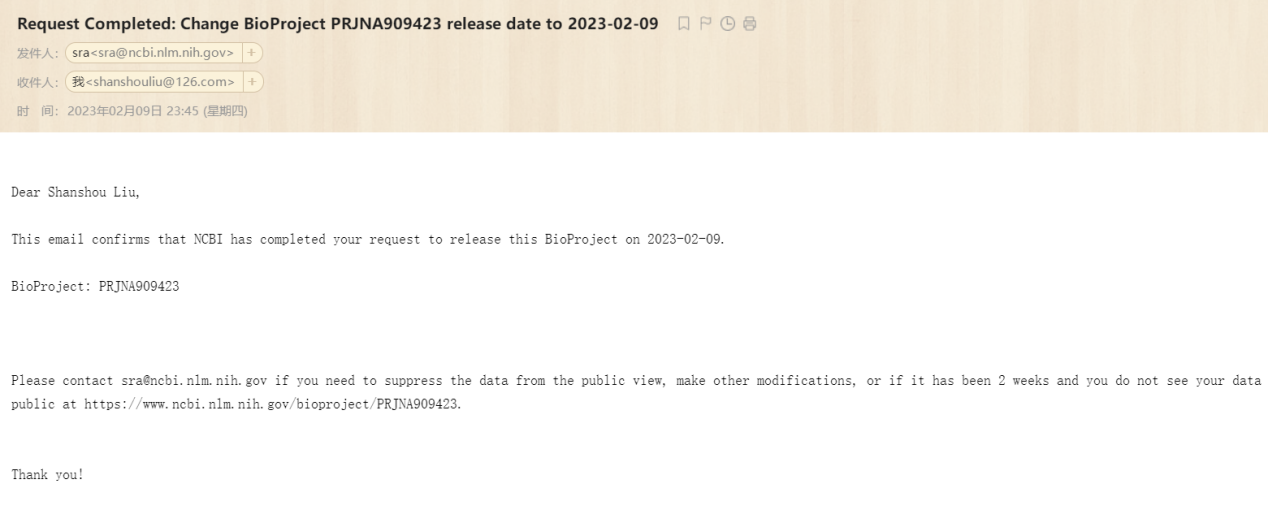

Supplement: Supplementary file 2 [file Image_2.tif]
