# Supplementary material for: Alteration of gut microbiota after heat acclimation may reduce organ damage by regulating immune factors during heat stress
Source: Front Microbiol. 2023 Feb 23;14:1114233. doi: 10.3389/fmicb.2023.1114233 (PMC9995595; doi:10.3389/fmicb.2023.1114233)
Supplement: Supplementary file 3 [file Presentation_1.PDF]

Supplementary Material

Alteration of gut microbiota after heat acclimation may reduce organ damage by regulating immune factors during heat stress

Shanshou Liu[1#], Dongqing Wen[2#], Chongyang Feng[1], Chaoping Yu[1], Zhao Gu[2], Liping Wang[2], Zhixiang Zhang[1], Wenpeng Li[1], Shuwen Wu[1], Yitian Liu[1], Chujun Duan[1], Ran Zhuang[1]*, Lihao Xue[2]*

*** Correspondence:** Ran Zhuang: fmmuzhr@fmmu.edu.cn; Lihao Xue: Xuelihao@fmmu.edu.cn

1 Supplementary Figures

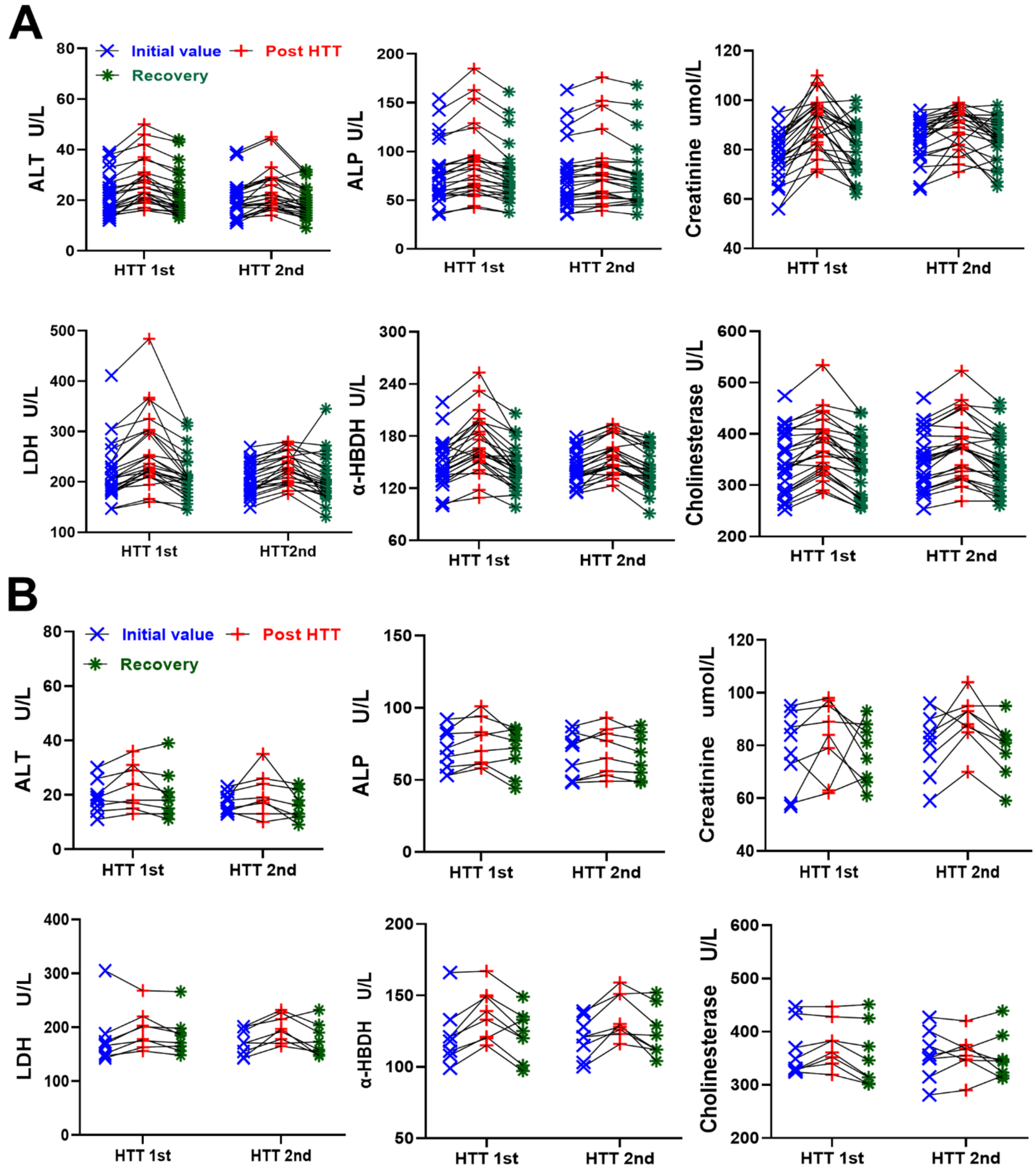

Supplementary Figure 1. Changes in plasma levels of organ function biomarkers in participants before and after HTT. **A.** Training group (n = 24). **B.** Control group (n = 8).

Abbreviations: ALT, alanine aminotransferase; ALP, alkaline phosphatase; LDH, lactic dehydrogenase; a-HBDH, alpha-hydroxybutyric dehydrogenase.

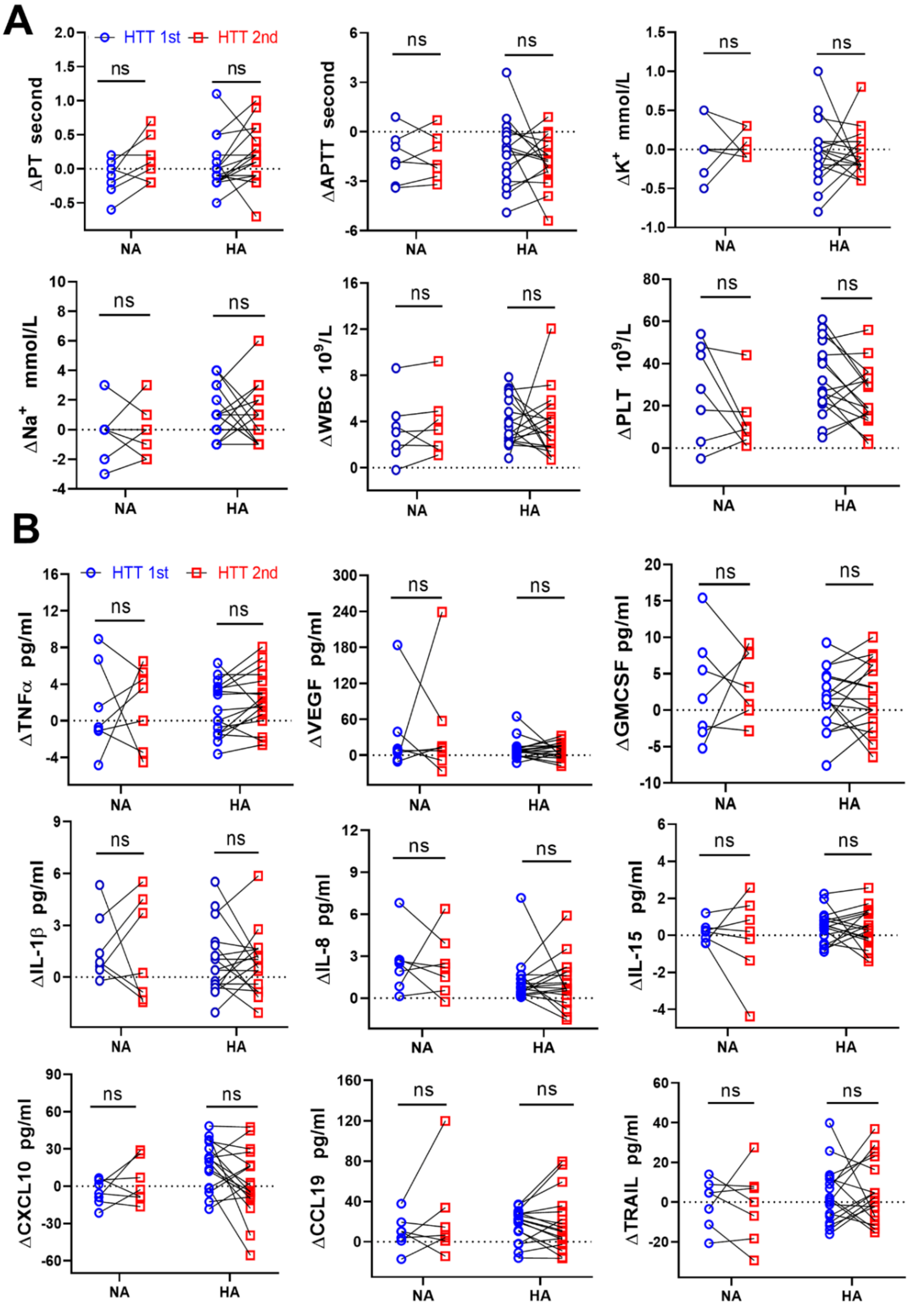

Supplementary Figure 2. Molecules with no significant difference in plasma content of the HA and NA groups before and after training. **A.** Laboratory results: PT and APTT (coagulation function); K^+ and Na^+ (electrolyte concentrations); WBC and PLT (peripheral blood cell content). **B.** Luminex

results: TNF-α, VEGF, and GMCSF (cytokine); IL-1β, IL-8, and IL-15 (inflammatory factor); CXCL10 and CCL19 (chemokine); TRAIL (tumor necrosis factor). HA n = 17; NA n = 7; ns, No significance.

Abbreviations: PT, prothrombin time; APTT, activated partial prothrombin time; WBC, white blood cell; PLT, platelet; TNF-α, alpha-tumor necrosis factor; VEGF, vascular endothelial growth factor; GMCSF, granulocyte-macrophage colony stimulating factor; TRAIL, TNF-related apoptosis inducing-ligand.

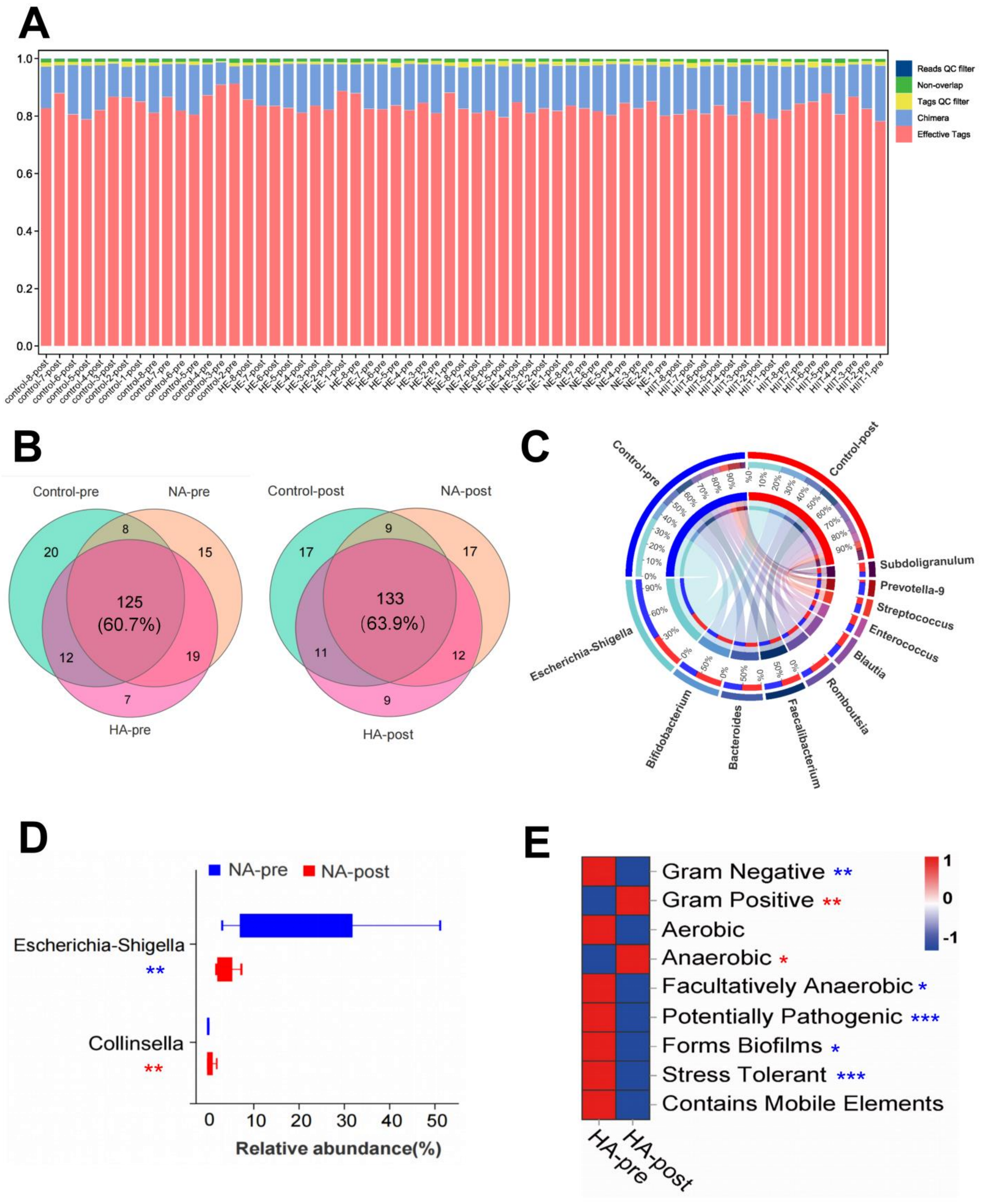

Supplementary Figure 3. Sequencing results of the gut microbiota of the participants. **A.** Results of sample quality control indicate that the average effective data ratio exceeds 80%. **B.** Venn plot of the number of gut microbial species (genus) before and after HAT. **C.** Circos diagram showing no significant difference in the proportion of Top 10 bacteria in the control group. **D.** Wilcoxon rank sum test was used to analyze the difference in the proportion of bacteria before and after training in the NA groups. In the NA group, Escherichia-Shigella, a potential pathogenic strains, was

significantly reduced. However, Collinsella increased significantly. E. The proportion of 7 functional phenotype bacteria changed significantly after acclimation, among which potential pathogenic bacteria decreased. HA n = 17; NA n = 7; Control n = 7.

2 Supplementary Table

Supplementary Table 1 The value assignment of different training elements

Team	Exercise time	Exercise intensity	Temp & Humidity
**NE-post**	H (2 hours/d)	M (HR 120~150 bpm)	M (T 30 ℃ H 50%)
**HIIT-post**	M (1 hour/d)	H (HR >150 bpm)	H (T 35 ℃ H 75%)
**HE-post**	H (2 hours/d)	M (HR 120~150 bpm)	H (T 35 ℃ H 75%)
**NE, HIIT, HE-pre**	L (0)	L (HR <100bpm)	L (T 25 ℃ H 40%)

Abbreviations: L, low; M, moderate; H, high.
